# Mapping default mode connectivity alterations following a single season of subconcussive impact exposure in youth football

**DOI:** 10.1002/hbm.25384

**Published:** 2021-03-18

**Authors:** Jesse C. DeSimone, Elizabeth M. Davenport, Jillian Urban, Yin Xi, James M. Holcomb, Mireille E. Kelley, Christopher T. Whitlow, Alexander K. Powers, Joel D. Stitzel, Joseph A. Maldjian

**Affiliations:** ^1^ Advanced Neuroscience Imaging Research (ANSIR) Laboratory University of Texas Southwestern Medical Center Dallas Texas USA; ^2^ Department of Radiology University of Texas Southwestern Medical Center Dallas Texas USA; ^3^ Department of Biomedical Engineering Wake Forest School of Medicine Winston Salem North Carolina USA; ^4^ Virginia Tech – Wake Forest School of Biomedical Engineering Wake Forest School of Medicine Winston Salem North Carolina USA; ^5^ Department of Radiology – Neuroradiology Wake Forest School of Medicine Winston Salem North Carolina USA; ^6^ Clinical and Translational Sciences Institute Wake Forest School of Medicine Winston Salem North Carolina USA; ^7^ Department of Neurosurgery Wake Forest School of Medicine Winston Salem North Carolina USA; ^8^ Childress Institute for Pediatric Trauma Wake Forest School of Medicine Winston Salem North Carolina USA

**Keywords:** concussion, connectivity, fMRI, football, subconcussion, youth

## Abstract

Repetitive head impact (RHI) exposure in collision sports may contribute to adverse neurological outcomes in former players. In contrast to a concussion, or mild traumatic brain injury, “subconcussive” RHIs represent a more frequent and asymptomatic form of exposure. The neural network‐level signatures characterizing subconcussive RHIs in youth collision‐sport cohorts such as American Football are not known. Here, we used resting‐state functional MRI to examine default mode network (DMN) functional connectivity (FC) following a single football season in youth players (*n* = 50, ages 8–14) without concussion. Football players demonstrated reduced FC across widespread DMN regions compared with non‐collision sport controls at postseason but not preseason. In a subsample from the original cohort (*n* = 17), players revealed a negative change in FC between preseason and postseason and a positive and compensatory change in FC during the offseason across the majority of DMN regions. Lastly, significant FC changes, including between preseason and postseason and between in‐ and off‐season, were specific to players at the upper end of the head impact frequency distribution. These findings represent initial evidence of network‐level FC abnormalities following repetitive, non‐concussive RHIs in youth football. Furthermore, the number of subconcussive RHIs proved to be a key factor influencing DMN FC.

## Graphical abstract

Using resting‐state functional MRI, DeSimone and colleagues demonstrate transient network‐level functional connectivity abnormalities of the default mode network following repetitive, non‐concussive head impact exposure over the course of a single youth football season.

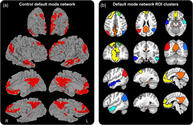

## 1 INTRODUCTION

Repetitive head impacts (RHI) in collision sports, such as American Football, may contribute to adverse long‐term neurological outcomes (Baugh et al., 2012; Gavett, Stern, & McKee, 2011; Huber, Alosco, Stein, & McKee, 2016; McKee et al., 2009; McKee et al., 2013; McKee, Alosco, & Huber, 2016; Montenigro et al., 2017; Stern et al., 2011). RHIs may encompass a combination of symptomatic mild traumatic brain injury (mTBI), or concussion, as well as more frequent, asymptomatic “subconcussive” impacts (Bailes, Petraglia, Omalu, Nauman, & Talavage, 2013). The frequent and repetitive nature of these so‐called subconcussive head impacts may have more detrimental effects on brain function and neuropathology (Tagge et al., 2018). However, the effects of subconcussive RHIs on the youth brain remain poorly understood. At the youth football level, players may sustain hundreds of subconcussive head impacts over the course of a single season, many at high‐magnitude forces on the order of high‐school and collegiate levels (Cobb et al., 2013; Daniel, Rowson, & Duma, 2012; Kelley et al., 2017; Urban et al., 2019). Early exposure to RHIs in tackle football during a high brain maturation period (≤ 12 years) may be a key contributing factor to later‐life neurobehavioral decline, especially with cumulative exposure over the course of a playing career (Alosco et al., 2018; Stamm et al., 2015).

A deeper understanding of the neural mechanisms underlying subconcussive RHI exposure is central to refining sport safety policies and developing targeted interventions to counteract adverse neurological events. Functional connectivity (FC), based on the synchronicity of spontaneous blood‐oxygen‐level dependent (BOLD) signal during resting‐state functional MRI (rsfMRI), is advantageous for detecting changes in functional brain architecture (Biswal, Yetkin, Haughton, & Hyde, 1995; Fox & Raichle, 2007). The default mode network (DMN) (Buckner, Andrews‐Hanna, & Schacter, 2008; Raichle, 2015; Raichle et al., 2001), a spatially distributed set of cortical regions involved in the mediation of task‐independent mental states, is amongst the most extensively studied resting‐state networks (RSN) with high test–retest reliability (Cole, Pathak, & Schneider, 2010; Shehzad et al., 2009). Using rsfMRI, greater emphasis has been placed on understanding the network‐level connectivity changes of the DMN following concussion (Borich, Babul, Yuan, Boyd, & Virji‐Babul, 2015; Czerniak et al., 2015; Iraji et al., 2015; Johnson et al., 2012; Mayer, Mannell, Ling, Gasparovic, & Yeo, 2011; Militana et al., 2016; Slobounov et al., 2011; Zhou et al., 2012; Zhu et al., 2015). An emerging body of work has pointed to abnormal DMN FC outcomes following repetitive, non‐concussive head impact exposure in high‐school and collegiate collision‐sport cohorts (Abbas et al., 2015; Abbas et al., 2015; Johnson, Neuberger, Gay, Hallett, & Slobounov, 2014; Manning et al., 2020; Reynolds et al., 2018; Slobounov et al., 2017). However, an understanding of the underlying changes in network‐level connectivity of the DMN following subconcussive impact exposure at the youth level is not yet clear.

To address this issue, we performed a prospective and longitudinal cohort study to examine changes in rsfMRI FC of the DMN in youth tackle football players (*n* = 50; ages 8–14 years). Employing seed‐based correlation and a combination of voxel‐wise and region‐of‐interest (ROI) approaches, the primary aim was to test the hypothesis that football‐related exposure to subconcussive RHIs over the course of a single season leads to aberrant network‐level connectivity of the DMN relative to healthy non‐collision sport controls. Second, we performed an exploratory analysis contrasting in‐ and off‐season changes in DMN FC in football player subsample (*n* = 17) with seven‐month follow‐up data availability (i.e., beginning of the subsequent season). Lastly, we tested the hypothesis that a higher number of experienced head impacts is associated with more adverse FC outcomes in football players.

## 2 METHODS

### 2.1 Participants

**FIGURE 1:**
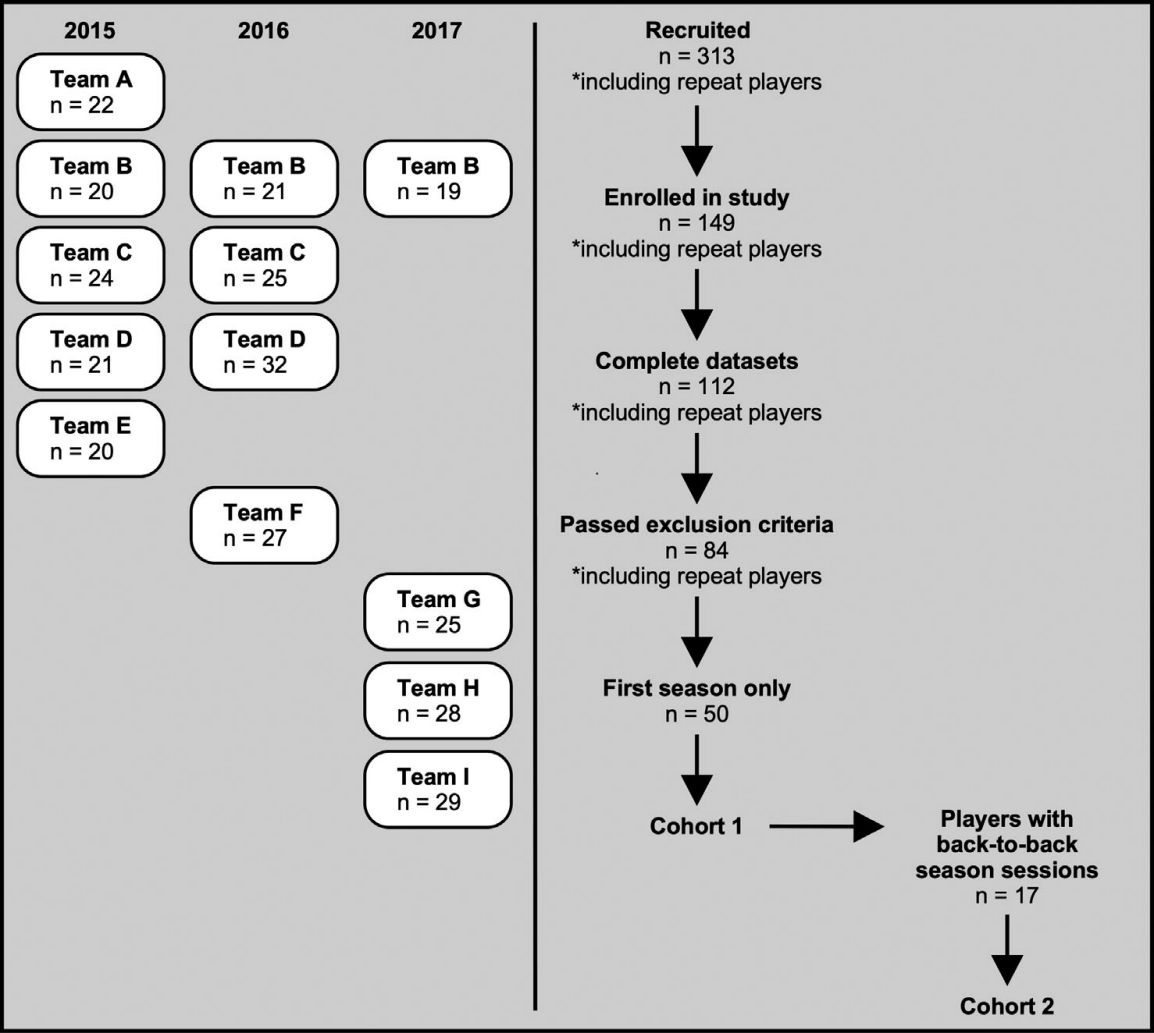
Flowchart of study enrollment. The left panel shows the number of teams and respective players who were recruited for the study between 2015 and 2017. The right panel describes the process of recruitment to final enrollment for Cohorts 1 and 2

Football players were recruited from nine local youth football teams in Forsyth County, NC between 2015 and 2017. Four to five teams were selected to participate each year. A trained research technician was present at all practice and game sessions for the acquisition of head impact sensor and video data. During recruitment, study staff attended pre‐season parent/player meetings and presented study objectives. A total of 313 players across nine teams were recruited for participation. Of the 313 total players, 149 elected to enroll in the study. A total of 112 players completed both preseason and postseason MRI acquisition sessions and the remaining 37 were excluded. An additional set of 28 players were removed due to enrollment exclusion criteria, which included self/parent‐reported history of neurological and/or psychiatric illness, concussion within the last year, and MRI contraindication (i.e., motion and susceptibility artifacts). Of the remaining 84 pairs of the preseason and postseason scans, 34 included sessions by the same player across multiple seasons. We opted to only use the first enrollment season to limit the inclusion of multiple sessions by the same player. A total of 50 male football players were ultimately retained for study inclusion (average age = 11.5 [*SD* = 1.2] years) (Figure 1). Of note, of the 50 total players, 17 had availability of multiple MRI acquisition sessions across back‐to‐back seasons. These player scans were retained for an exploratory analysis and the basis for this analysis is described in Section 2.3. Two football players included in the study had self/parent‐reported history of a single concussion more than a year prior to the season and none were diagnosed with a concussion during the season.

Youth male control athletes with no prior tackle football experience were also recruited for this study. Controls were recruited from local non‐collision sports teams during a scheduled player/parent meeting. Control participants were represented across six different sports (seven basketball, seven baseballs, two soccer, two tennis, one swimming, one karate). Of the 33 control athletes who elected to enroll in the study, 20 (average age = 11.4 [*SD* = 1.2] years) were retained for study inclusion based on the aforementioned exclusion criteria. Demographic variables for football and control participants are presented in Table S1.

This study was conducted in compliance with the Health Insurance Portability and Accountability Act. All participants and their parental guardians provided signed assent and consent, respectively, and were in full understanding of experimental objectives. All experimental procedures were approved and monitored by the Wake Forest School of Medicine Institutional Review Board Committee and conducted in ethical accord with the Declaration of Helsinki.

### 2.2 Head impact telemetry system data acquisition

Football participants were fitted with helmet‐based accelerometers using the Head Impact Telemetry System (HIT System: Simbex, Lebanon, NH) (Beckwith, Greenwald, & Chu, 2012; Crisco, Chu, & Greenwald, 2004) for real‐time acquisition of head impact kinematics during all practice and game sessions. Only those players who were enrolled in the study were equipped with the HIT System. Only players with a head circumference that could adequately fit a medium or large size Riddell Speed® or Riddell Revolution® helmet were permitted to participate, as the head impact sensor system was not compatible with smaller helmet sizes. The HIT System measures the location and magnitude of head impacts using an array of six spring‐mounted single‐axis accelerometers in contact with the head surface. Data acquisition was initiated upon detection of peak resultant linear acceleration of the head above 10 *g*. An important consideration is that indirect head impacts (e.g., direct impacts to the torso) can initiate data acquisition due to the associated change in peak resultant acceleration of the head above this threshold. All games and practices were video recorded and any accelerations due to activities other than head impacts (e.g., dropped helmet) were excluded from the analysis. For the current study, we calculated the total number of head impacts, head impact severity based on percentiles calculated from the distribution of linear (measured in *g*) and rotational acceleration (measured in rad/s^2^), and risk‐weighted exposure (RWE_CP_). RWE_CP_ represents a measure of cumulative head impact exposure encompassing the number and severity of experienced head impacts, in which the peak resultant linear and rotational accelerations of each impact are non‐linearly weighted using the combined probability risk function and summed for each player over the course of the season (Rowson et al., 2012; Rowson & Duma, 2013; Urban et al., 2013).

### 2.3 MRI acquisition and processing

All 50 football players included in this study were scanned at two separate time points, including up to 1 month preceding (i.e., preseason) and following (i.e., postseason) the season. The average time between preseason and postseason acquisition sessions was 4.7 (*SD* = 0.8) months. This sample of 50 players is henceforth referred to as Cohort 1. Players were asked to enroll across multiple seasons if they remained on a team selected to participate in the study. Of the 50 players who were included in the study, a subsample (*n* = 17) was scanned during the subsequent season. This provided the opportunity to examine, on an exploratory basis, DMN FC dynamics between the postseason acquisition scan of the first enrollment season and the preseason acquisition scan of the subsequent season (i.e., off‐season). The average time between these MRI acquisition sessions was 7.2 (*SD* = 0.6) months. This subsample of 17 players, representing four different teams, is henceforth referred to as Cohort 2. Control participants were scanned at two‐time points an average of 4.0 (*SD* = 0.9) months apart. The timing of initial and follow‐up acquisition scans did not always coincide with the preseason and postseason of their respective sport.

MRI sequences were acquired on a 3 Tesla Siemens Skyra MRI scanner using a 32‐channel head/neck radiofrequency coil (Siemens Medical, Erlangen, Germany). Participants laid supine and were instructed to remain still with their eyes closed and free of external thought for the experimental duration. The rsfMRI data were acquired using a gradient EPI sequence with the following parameters: repetition time (TR) = 2,000 ms, repetitions = 190, echo time (TE) = 25 ms, flip angle = 90°, field of view (FOV) = 61 × 64, 3.5 mm^3^ isotropic resolution. Structural T1‐weighted images were acquired for spatial normalization using a 3D‐MPRAGE sequence with the following parameters: TR = 2,300 ms, TE = 2.98 ms, flip angle = 90°, FOV = 256 × 256 mm, 1 mm^3^ isotropic resolution.

### 2.4 Functional MRI data preprocessing

Processing of rsfMRI data was carried out using *afni_proc.py* in Analysis of Functional NeuroImages (AFNI: Version 13.0.3). Preprocessing for each scan included removal of three base volumes to account for scanner magnetization equilibrium, de‐spiking of extreme time‐series outliers, acquisition‐dependent slice‐timing correction, and rigid‐body volume registration. Anatomical and functional images were co‐registered, skull stripped, and non‐linearly warped to the MNI avg152 template with a resampled isotropic resolution of 2 mm. Functional volumes were spatially smoothed to improve the signal‐to‐noise ratio using a 4 mm Gaussian FWHM kernel. Nonspecific or spurious sources of variance from the BOLD time series, including six head motion derivatives describing rigid‐body transformations, as well as mean global, CSF, and local white matter signals, were regressed. The regression step also included censoring of consecutive functional volumes >0.5 mm in relative motion and time points with >10% of total voxels identified as signal outliers. Across pre‐ and postseason scans the average number of censored volumes was 11.62 (*SD* = 12.21, 3.1% of total TRs) and 11.35 (*SD* = 15.12, 3.03% of total TRs) for football and control participants, respectively. Lastly, the BOLD time series data was bandpass filtered between 0.008 and 0.1 Hz.

### 2.5 Functional connectivity statistical analysis

A seed‐based correlation approach was used to examine FC using an *a priori* selected spherical seed region within the posterior cingulate cortex (PCC [MNI coordinates: 0, −53, 25; radius = 6 mm]), a central node of the DMN (Fox et al., 2005; Greicius, Krasnow, Reiss, & Menon, 2003). For each residual time series (preseason, postseason), Pearson correlation coefficients were computed between the seed time series and that of all other voxels and converted to Z‐scores using the Fisher r‐to‐z transformation. Preseason Z‐score maps for each participant were subtracted from their postseason counterpart to compute in‐season delta FC maps.

**FIGURE 2:**
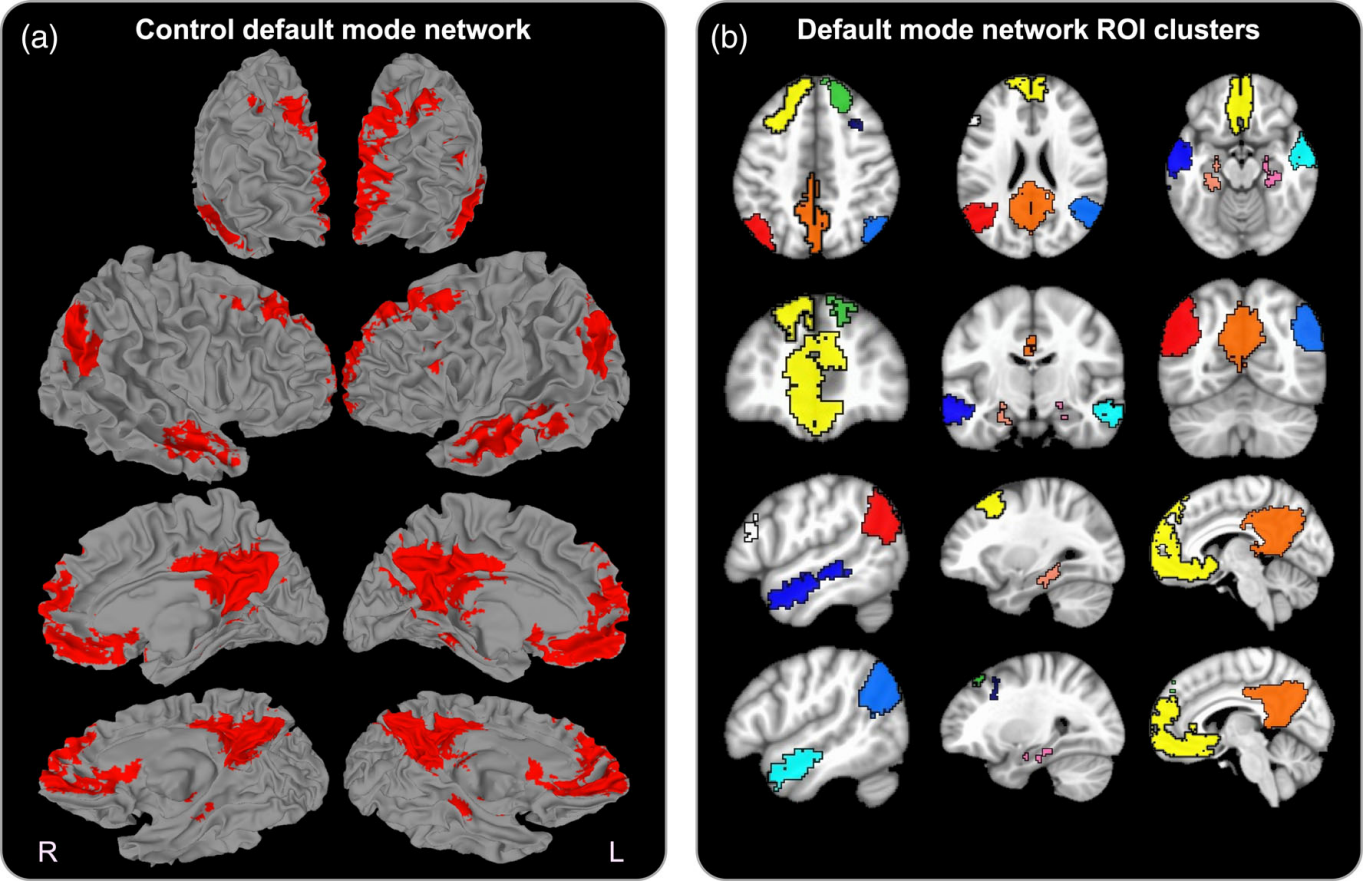
Control default mode network and ROI clusters. (a) Three‐dimensional rendering of the default mode network (red) comprising voxels that exhibit a significant positive time‐series correlation with that of the seed region in the posterior cingulate cortex for control participants (*p* < .05 FWER‐corrected). (b) Axial, sagittal, and coronal color‐coded depiction of architectural hubs comprising the FWER‐corrected default mode network used in the ROI analysis. PFC/l‐DFC (yellow), PCC/precuneus (orange), l‐TPC (red), r‐TPC (light blue), r‐DFC (lime green), r‐MFG (navy), l‐IFG (white), l‐LTC (dark blue), r‐LTC (cyan), l‐PHF I and II (salmon), and r‐PHF (pink)

Group‐level analyses were anatomically constrained to regions comprising the DMN using a masking procedure derived from control participants. To this end, voxels that demonstrated a positive time series correlation with the seed location (*p* < .05 uncorrected) were obtained across control participants, separately for both preseason and postseason scans. Positive correlation maps were then concatenated across scans and averaged across control participants to generate a single uncorrected DMN mask. Positive cerebellar correlations were discarded due to variable and incomplete spatial coverage across participants. To refine the mask, a one‐sample *t* test in AFNI with the *Clustsim* option was performed on control datasets at preseason. The *Clustsim* option provides the cluster‐extent volume required to limit false‐positive correlations with the seed location. This option advised a cluster‐extent volume ≥ 400 mm^3^, equivalent to *p* < .005 (*p* < .05 FWER‐corrected). Positive correlation maps were then re‐obtained using the advised cluster‐extent threshold, concatenated across scans, and averaged across participants. As shown in Figure 2a, the resultant mask is in agreement with previous literature, comprising established DMN regions that exhibit positive time‐series correlations with the PCC under task‐negative conditions (Fox et al., 2005; Greicius et al., 2003).

Group‐level DMN FC differences between Cohort 1 football players and controls were examined using two separate approaches. In the first analysis, voxel‐wise independent‐sample t‐tests in AFNI (*3dttest++*) were used to compare FC maps, separately for preseason, postseason, and in‐season delta. Age and BMI were entered as covariates for preseason and postseason tests and time between acquisition sessions was entered as an additional covariate when comparing delta maps. Group‐level effects were considered significant at a minimum cluster volume of 400 mm^3^ (*p* < .05 FWER‐corrected), in accordance with the aforementioned *Clustsim* output.

A drawback of the voxel‐wise approach is that test statistics are computed across thousands of voxels and significant group‐level effects depend on a rigorous cluster‐extent threshold in accounting for multiple comparisons. Limiting statistical tests to a condensed set of defined regions is advantageous in reducing the severity of the alpha level adjustment for multiple test statistics (Poldrack, 2007). To this end, a region‐of‐interest (ROI) approach was used in a second analysis to examine group differences in mean Z‐score values within subregions of the DMN. In this approach, clustered voxels from the FWER‐corrected DMN mask with a minimum cluster‐extent volume of 400 mm^3^ were delineated *a posteriori* and used as individual ROIs to compute mean preseason and postseason for each participant. As shown in Figure 2b, this approach yielded 12 individual ROIs comprising the ventromedial/medial prefrontal cortex and left dorsal frontal cortex (PFC/l‐DFC), right dorsal frontal cortex (r‐DFC), left inferior frontal gyrus (l‐IFG), right middle frontal gyrus (r‐MFG), PCC/precuneus, right/left temporoparietal cortex (r‐ and l‐TPC), right/left lateral temporal cortex (r‐ and l‐LTC), right parahippocampal formation (r‐PHF), and two separate clusters in the left parahippocampal formation (l‐PHF I/II). Preseason Z‐scores for each ROI and each participant were subtracted from their postseason counterpart to compute in‐season delta Z‐scores. For each time point (preseason, postseason, in‐season delta), group‐level differences in mean Z‐score values across all ROIs were examined using a multivariate analysis of covariance (MANCOVA) model in R (Version 4.0.0; R Core Team, 2020). Age and BMI were entered into the model as covariates. Delta comparisons included time between acquisition scans as an additional covariate. Significant multivariate group main effects were further decomposed using univariate tests across all ROIs and considered significant at *p* < .05 FDR‐corrected.

### 2.6 Longitudinal connectivity changes in football players

In Cohort 2 football players, we tested whether in‐season delta FC, representing a 4‐month period of RHI exposure, differed from that of off‐season delta FC, representing a 7‐month withdrawal from football‐related activity. A first step included the previous ROI analysis to identify group‐level differences between Cohort 2 football players and controls. The purpose of this first step was to determine whether Cohort 2 football players were representative of the group as a whole, as well as identify a subset of ROIs that best‐characterized football and control participants according to in‐season delta FC. Second, regions identified as displaying significant in‐season delta FC group effects in the ROI analysis were decomposed longitudinally in Cohort 2 football participants. To this end, postseason Z‐scores for each identified ROI and each participant were subtracted from their subsequent preseason counterpart scan to compute off‐season delta Z‐scores. Separately for each region, a two‐way linear mixed model was used to examine differences between in‐ and off‐season delta Z‐scores, with time (in‐season, off‐season) and participants as the within‐ and between‐subjects random factors, respectively. Age and BMI were included in the models as covariates. The main effects of time across ROIs were considered significant at *p* < .05 FDR‐corrected.

### 2.7 Effect of head impact frequency on connectivity outcomes in football players

In a final exploratory analysis, we sought to examine whether a high and the low number of cumulative head impacts in football players yielded differential FC outcomes. In Cohort 1, football participants were partitioned into low and high impact groups according to the lower and upper third percentiles, respectively, of the head impact frequency distribution across participants. This split resulted in 18 low impact players with head impact values ranging from 22 to 151 and 18 high impact players with head impact values ranging from 349 to 2016. Due to the small sample size in Cohort 2 and to preclude further player exclusion, low and high impact groups were partitioned according to the 50th percentile of the head impact frequency distribution (413 impacts). This split resulted in eight players in the low impact group with head impact values ranging from 43 to 341 and nine players in the high impact group with head impact values ranging from 413 to 2016. Players who experienced 413 impacts (i.e., 50th percentile) were included in the high impact group. Table 1 provides descriptive statistics of head impact kinematics based on each respective partition. Specifically, for Cohort 1, we examined whether the number of head impacts differentially influenced the change between preseason and postseason FC in a subset of ROIs that distinguished football and control participants according to significant postseason group effects. For Cohort 2, we examined whether the number of head impacts differentially influenced in‐ and off‐season delta FC in a subset of ROIs that distinguished football and control participants according to significant in‐season delta group effects. ROI Z‐scores were decomposed based on the number of impacts using a two‐way linear mixed model, with time and participants as the within‐ and between‐subjects factors, respectively. Age and BMI were included in the model as covariates. Separately for each cohort and impact allocation, the main effects of time across ROIs were considered significant at *p* < .05 FDR‐corrected.

**TABLE 1:** Descriptive statistics of head impact kinematics

All football players							
**Cohort 1**	**All players (*n* = 50)**						
**Variable**	**Mean**	**Median**	**95% confidence**				
Total number of head impacts	327.7	257	161–383				
Median peak linear acceleration (g)	18.9	18.3	17.5–18.9				
95th percentile linear acceleration (g)	51.7	49.7	46.6–52.5				
Median peak rotational acceleration (rad/s^2^)	928.3	925.5	901–948				
95th percentile rotational acceleration (rad/s^2^)	2,485	2,440	2,310–2,570				
RWE_CP_	0.53	0.19	0.13–0.35				
**High and low impact partitions**							
**Cohort 1**	**Low impact players (*n* = 18)**			**High impact players (*n* = 18)**			
**Variable**	**Mean**	**Median**	**95% confidence**	**Mean**	**Median**	**95% confidence**	***p*‐value (MW)**
Total number of head impacts	81.5	79	54.8–107	640.1	489.5	362–612	**< .0001**
Median peak linear acceleration (g)	18.2	17.8	17–18.5	19.8	19.7	17.9–21.3	**< .02**
95th percentile linear acceleration (g)	50.3	46.9	40.8–52.1	53.4	50.8	45.1–56	ns
Median peak rotational acceleration (rad/s^2^)	904.8	912	878–949	956.6	962.6	925–1,010	ns
95th percentile rotational acceleration (rad/s^2^)	2,616.6	2,466.7	2,120–2,730	2,492.6	2,534.6	2,350–2,770	ns
RWE_CP_	0.12	0.06	0.0003–0.11	0.92	0.54	0.14–0.88	**< .0001**
**Cohort 2**	**Low impact players (*n* = 8)**			**High impact players (*n* = 9)**			
**Variable**	**Mean**	**Median**	**95% confidence**	**Mean**	**Median**	**95% confidence**	***p*‐value (MW)**
Total number of head impacts	236.1	270.5	191–384	847.2	837	528–1,290	**< .001**
Median peak linear acceleration (g)	18.7	18.1	15.6–19.7	20.2	20.6	18.8–22.5	ns
95th percentile linear acceleration (g)	50.5	50.6	45.5–55.7	54.3	52	40.9–60.5	ns
Median peak rotational acceleration (rad/s^2^)	933.2	901.8	804–980	950.5	969.6	878–1,070	ns
95th percentile rotational acceleration (rad/s^2^)	2,343.5	2,274.4	2060–2,480	2,492.8	2,461.6	2,100–2,840	ns
RWE_CP_	0.53	0.29	0–0.48	1.16	0.55	0–1.25	ns

*Note*: Descriptive statistics of head impact kinematics for all football players (Cohort 1) not partitioned according to the number of head impacts (top), as well as Cohort 1 (middle) and 2 (bottom) football player partitions based on the cumulative number of experienced head impacts during the season. In Cohort 1, football players were partitioned into low and high impact groups based on the lower and upper third percentiles of the head impact frequency distribution, respectively. In Cohort 2, football players were partitioned according to the median of the head impact frequency distribution. Confidence intervals were computed at the 95% level on the median value for each metric using the bootstrap method (5,000 permutations). Median values for each metric were compared across low and high impact football players using Mann–Whitney U tests and considered significant at *p* < .05.

Abbreviations: MW, Mann–Whitney *U* test; ns, not statistically significant; RWE_CP_, combined probability risk‐weighted exposure.

## 3 RESULTS

### 3.1 Reduced DMN functional connectivity in youth football players

**FIGURE 3:**
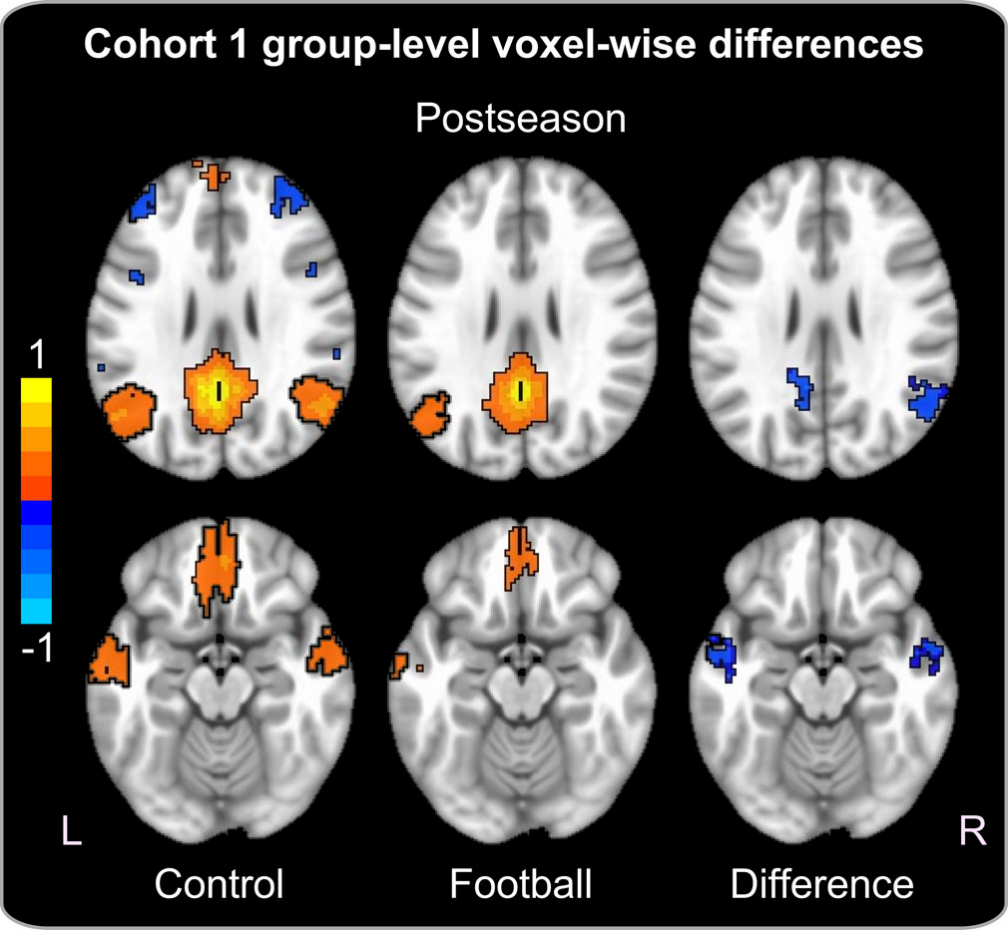
Cohort 1 group‐level voxel‐wise differences. Axial slice representation demonstrating significant postseason group‐level voxel‐wise differences in functional connectivity of the default mode network between control and Cohort 1 football participants. Group averaged Z‐score correlation maps in control and football participants are displayed in the left and center panels, respectively, whereas significant group‐level t‐statistic maps between control and football participants are displayed in the right panel. Red and blue voxels in the average Z‐score correlation maps demonstrate a significant positive and negative correlation with the posterior cingulate cortex seed, respectively, whereas the blue voxels in the t‐statistic maps represent significantly reduced functional connectivity in football players compared with controls. All maps are thresholded at *p* < .05 FWER‐corrected

In the voxel‐wise analysis (Figure 3), DMN FC did not differ between Cohort 1 football and control participants at preseason. At postseason, football participants demonstrated reduced FC compared with controls in four clusters comprising the r‐ and l‐LTC, the angular gyrus region of the r‐TPC, and left PCC/precuneus. Postseason group‐level effects were significant at *p* < .05 FWER‐corrected. No significant group‐level effects were revealed when comparing in‐season delta FC maps between control and football participants.

**FIGURE 4:**
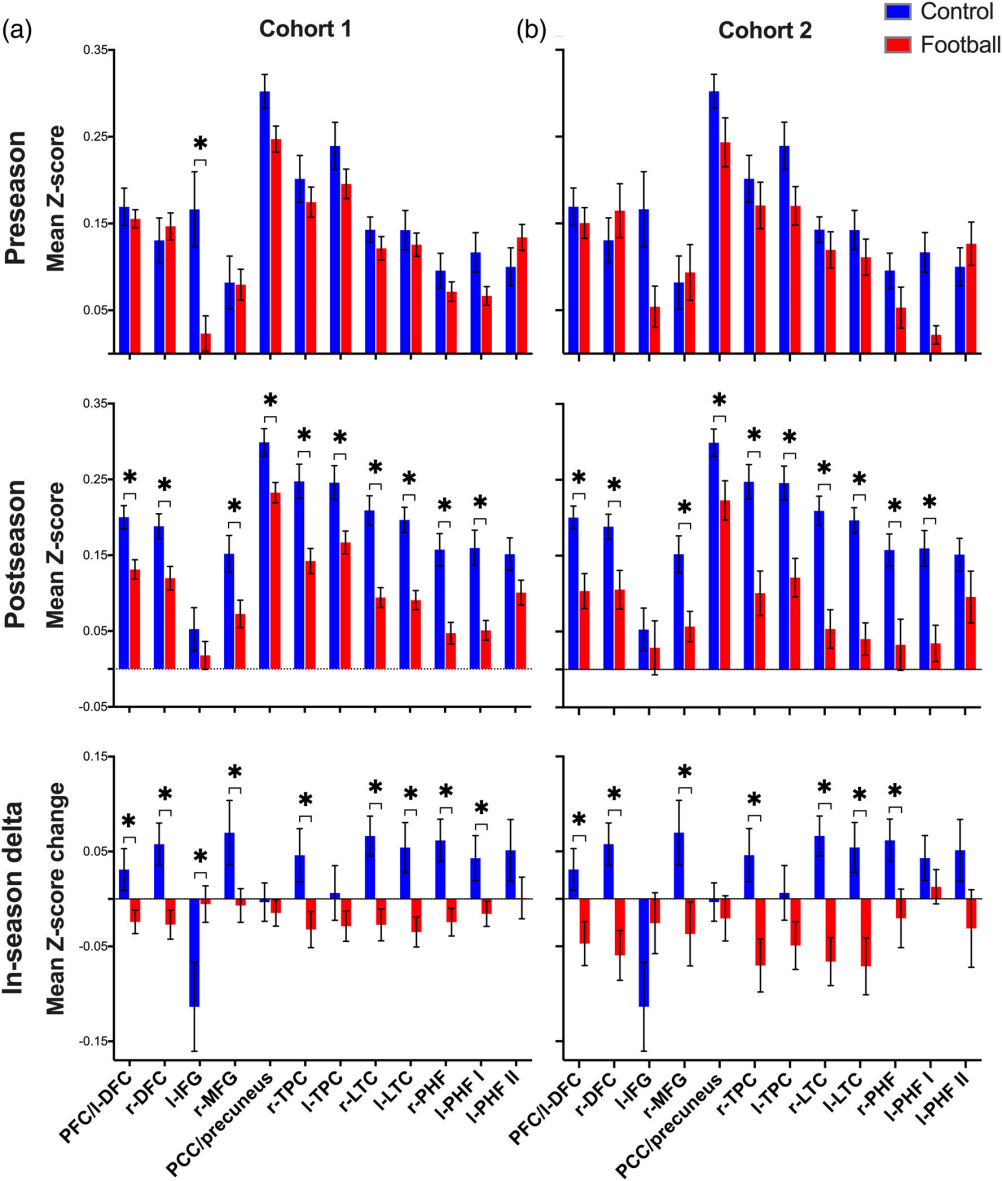
Region‐of‐interest functional connectivity. Grouped bar plots demonstrating group‐level differences in mean Z‐score values (± *SEM*) between controls (blue bars) and football players (red bars) in (a) Cohort 1 and (b) Cohort 2. The mean Z‐score values for preseason, postseason, and in‐season delta are shown in the upper, middle, and lower sub‐plots, respectively. Each panel displays a set of 12 a posteriori delineated ROIs of the default mode network (Figure 2b). Asterisks represent a significant group‐level difference between control and football participants (*p* < .05 FDR‐corrected)

A second analysis examined group‐level differences in FC within a set of 12 DMN ROIs using a MANCOVA model (Figure 4a). A significant group main effect was uncovered at preseason (F_12,55_ = 2.12, *p* = .03), indicating that mean Z‐score values in the l‐IFG were reduced in football compared with control participants (*p* < .05 FDR‐corrected). Results for postseason FC yielded a significant group main effect (F_12,55_ = 4.39, *p* = .00007), such that football players demonstrated reduced FC compared with controls across 10 of 12 ROIs, including the PFC/l‐DFC, r‐DFC, r‐MFG, PCC/precuneus, r‐ and l‐TPC, r‐ and l‐LTC, r‐PHF, and l‐PHF I (*p* < .05 FDR‐corrected). Results for in‐season delta FC yielded a significant group main effect (F_12,55_ = 2.98, *p* = .003), such that football players demonstrated a reduced change in mean Z‐score values between preseason and postseason, including the PFC/l‐DFC, r‐DFC, l‐IFG, r‐MFG, r‐TPC, r‐ and l‐LTC, r‐PHF, and l‐PHF I (*p* < .05 FDR‐corrected). Except for l‐IFG delta FC, which yielded a negative mean delta score in both groups, football and control participants demonstrated negative and positive mean delta scores across these ROIs, respectively. In other words, FC of the DMN became stronger during the 4‐month interscan interval in the control group but weaker during the in‐season months for football players.

### 3.2 Longitudinal connectivity changes in football players

We next examined longitudinal changes in FC across DMN ROIs in Cohort 2 football players with off‐season follow‐up data availability. A first step included an ROI analysis to examine group‐level differences between Cohort 2 football players and controls at preseason, postseason, and in‐season delta. As shown in Figure 4b, this analysis yielded consistent effects with the ROI analysis for Cohort 1 (Figure 4a), suggesting that the players in Cohort 2 were representative of the group as a whole. Significant group main effects were observed for the postseason (F_12,21_ = 2.98, *p* < .001) and in‐season delta (F_12,21_ = 2.58, *p* = .03) FC. Postseason FC was reduced in 10 of 12 ROIs in football players compared with controls (*p* < .05 FDR‐corrected). In‐season delta FC was reduced in seven of 12 ROIs in football compared with control participants (*p* < .05 FDR‐corrected), such that the change in FC across these ROIs was negative and positive in football and control participants, respectively.

**FIGURE 5:**
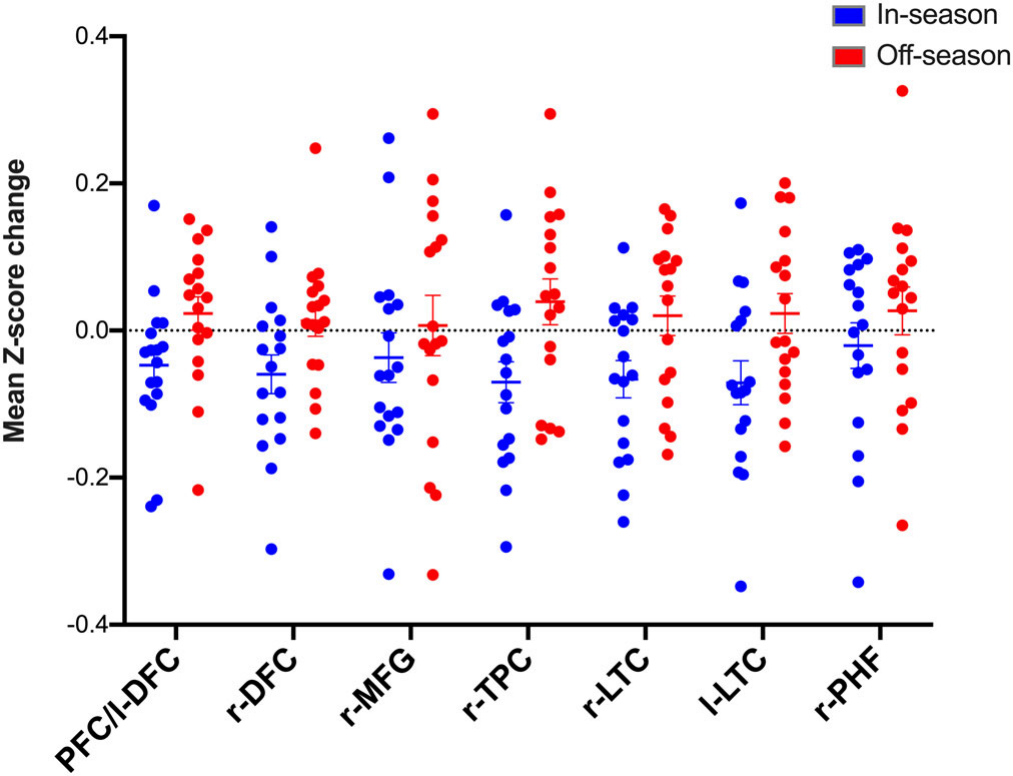
In‐ and off‐season functional connectivity delta. Grouped dot plots demonstrating the mean (± *SEM*) (horizontal lines) change in Z‐score values between preseason and postseason (i.e., in‐season delta, blue) and between postseason and follow‐up scans (i.e., off‐season delta, red) in Cohort 2 football participants. The x‐axis includes a set of seven ROIs derived from significant between‐group effects in the in‐season delta ROI analysis between control and Cohort 2 football participants (Figure 4b)

The seven regions identified as displaying significant group effects for in‐season delta FC in the ROI analysis were decomposed longitudinally in football players. As shown in Figure 5, in‐season delta Z‐scores yielded a negative mean across all ROIs, whereas off‐season delta Z‐scores yielded a positive mean. Five ROIs, including the PFC/l‐DFC, r‐DFC, r‐TPC, and r and l‐LTC demonstrated a trend toward significantly increased off‐season delta FC relative to in‐season (*p* = .064 FDR‐corrected). However, no significant main effects of time were observed following FDR correction, indicating that in‐season and off‐season delta FC did not reliably differ across these regions.

### 3.3 Head impact frequency differentially influences connectivity outcomes

**FIGURE 6:**
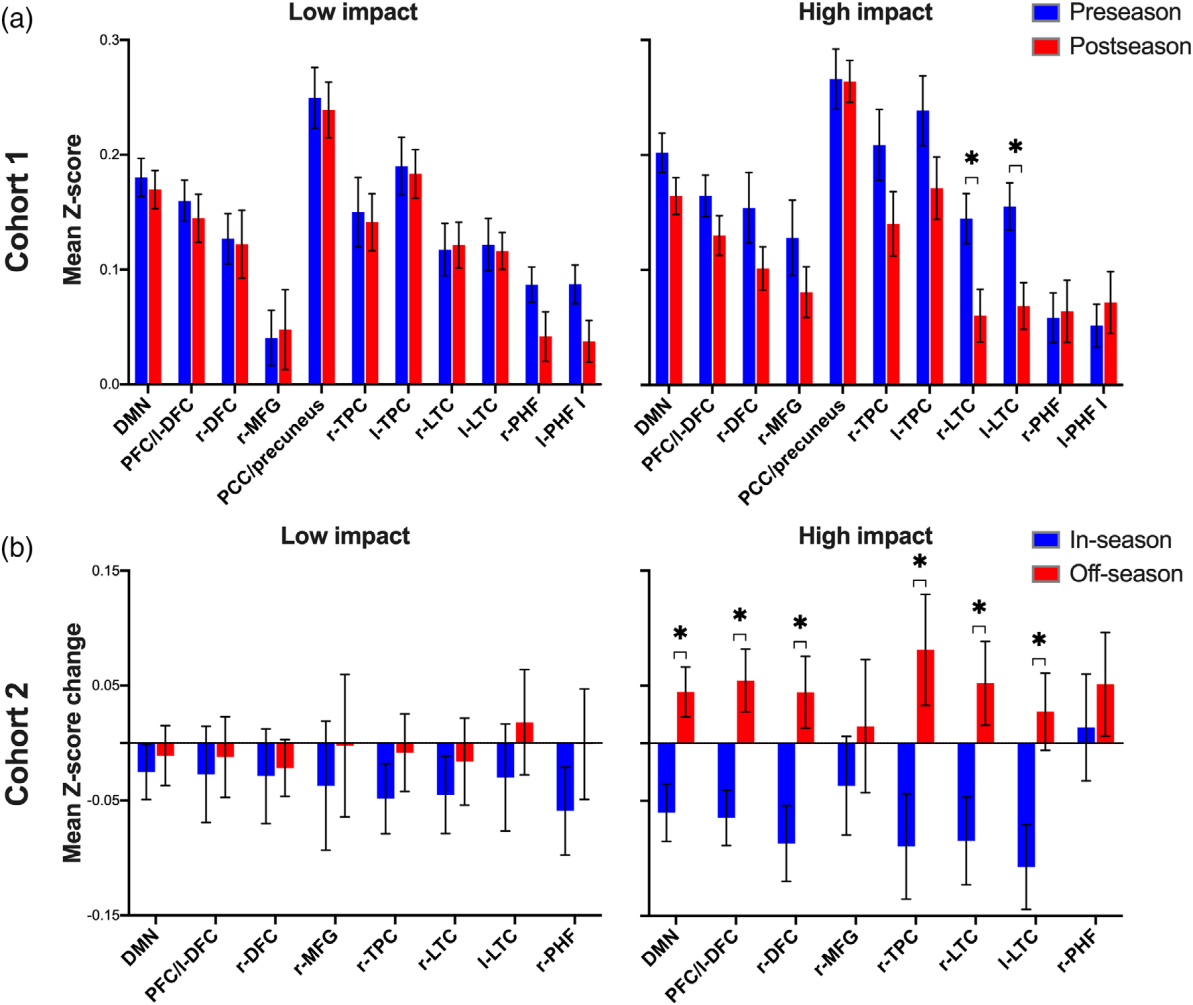
Head impact frequency and default mode connectivity. Grouped bar plots demonstrating the mean (± SEM) Z‐score values for (a) preseason and postseason in Cohort 1 football participants and (b) in‐ and off‐season delta in Cohort 2 football participants. For Cohort 1, the x‐axis denotes a set of 10 ROIs that characterized football and control participants according to group‐level effects (football vs. control) in the postseason ROI analysis, as well as the global default mode network (DMN). For Cohort 2, the x‐axis denotes a set of seven ROIs that characterized football and control participants according to group‐level effects (football vs. control) in the in‐season delta FC ROI analysis, as well as the global DMN. The left and right subplots depict Z‐scores obtained from players in the low and high impact frequency partitions for each football cohort, respectively. Asterisks represent a significant time main effect (*p* < .05 FDR‐corrected)

A linear mixed model was used to examine whether a high and low total number of cumulative head impacts over the course of the season differentially influenced FC outcomes. In the first analysis (Figure 6a), we examined whether a high and the low number of impacts differentially influenced the change between preseason and postseason FC in Cohort 1 football players. The 10 ROIs identified as displaying significant group effects at postseason (Figure 4a), as well as the global DMN were decomposed according to impact exposure. No significant main effects of time were uncovered for low impact players, indicating that preseason and postseason FC did not differ across these regions. The analysis yielded significant time effects for high impact players, such that mean postseason Z‐scores in the r and l‐LTC were reduced relative to preseason (*p* < .05 FDR‐corrected). Several other ROIs, including the r and l‐TPC, PFC/l‐DFC, r‐DFC, and r‐MFG, as well as the global DMN showed a trend of reduced postseason FC relative to preseason for high impact players (*p* < .1 FDR‐corrected).

In the second analysis (Figure 6b), we examined whether a high and low total number of head impacts differentially influenced in‐ and off‐season delta FC in Cohort 2 football participants. The seven ROIs identified as displaying significant in‐season delta FC between‐group effects (Figure 4b), as well as the global DMN were decomposed according to impact exposure. No significant main effects of time were uncovered for low impact players, indicating that in‐ and off‐season delta FC did not differ in these regions. Conversely, high impact players demonstrated significant time effects for PFC/l‐DFC, r‐DFC, r‐TPC, r‐ and l‐LTC, and the global DMN, such that the positive off‐season delta FC mean was increased compared with the negative in‐season delta FC mean (*p* < .05 FDR‐corrected).

## 4 DISCUSSION

This study represents an original report of the underlying FC changes characterizing cumulative exposure to subconcussive head impacts in youth tackle football players. The primary objective was to determine whether a single season of youth football in the absence of concussion imparts changes in DMN FC compared with a non‐collision sport control group. Football players demonstrated network‐level changes in FC across widespread hubs of the DMN at the postseason in both the voxel‐wise and ROI‐based analyses. A secondary aim was to examine longitudinal changes in DMN FC during in‐ and off‐season months in a subsample of football players with imaging data availability at a 7‐month follow‐up time‐point. In a subset of ROIs, football players demonstrated a negative in‐season delta FC but a positive, compensatory off‐season delta FC. A final aim was to examine whether the number of head impacts over the course of the season differentially influenced the change in DMN FC between preseason and postseason, as well as the change in delta DMN FC between in‐ and offseason months. Significant changes in DMN FC between preseason and postseason, as well as between in‐ and off‐season delta were specific to football players at the upper end of the head impact frequency distribution.

### 4.1 Reduced DMN functional connectivity in youth football players

Football players in Cohort 1 revealed reduced FC of widespread DMN regions at postseason, but not preseason, compared with non‐collision sport controls. Specifically, football players demonstrated reduced FC between the PCC and four clusters in the voxel‐wise analysis, including the PCC/precuneus, r‐TPC, and r‐ and l‐LTC (Figure 3). In the postseason ROI analysis, football players demonstrated reduced FC across all regions except for the l‐IFG and l‐PHF II (Figure 4a). In the ROI analysis, in‐season delta FC for football players was reduced in all regions except for the PCC/precuneus, l‐TPC, and l‐PHF II (Figure 4a). Except for a single region (l‐IFG), which demonstrated a negative FC change between preseason and postseason in both groups, the widespread in‐season delta FC group‐level effects were driven by a positive and negative delta score for control and football participants, respectively. In other words, FC of the DMN became stronger during the 4‐month interscan period in the control group but weaker during the 4‐month interscan period for football players.

Reduced connectivity between the PCC and DMN regions conforms to previous studies following mTBI. In players from multiple sports, Johnson et al. (2012) reported a reduced number of overall connections during the sub‐acute injury phase between the PCC and lateral parietal areas, as well as no significant connections between the PCC and afferents in the dorsolateral prefrontal and parahippocampal regions. Mayer et al. (2011) demonstrated reduced seed‐based FC of the PCC with the supramarginal gyrus and superior frontal gyrus during the sub‐acute phase following mTBI (Mayer et al., 2011). Zhu et al. (2015) reported reduced DMN connections within the superior frontal, hippocampal, and angular regions compared with controls 7 days following mTBI. In another study, Iraji et al. (2015) demonstrated reduced seed‐based FC between the PCC and precuneus but also stronger FC between the PCC and long‐range extra‐DMN afferents in the frontal lobe. The transient decrease in FC strength between the PCC and DMN nodes reported here also contradicts rsfMRI literature demonstrating increased DMN FC following TBI at varying severities (Han, Chapman, & Krawczyk, 2016; Hillary et al., 2014; Hillary et al., 2015; Iraji et al., 2016; van der Horn et al., 2017). As described by Hillary et al. (2014), patterns of hyperconnectivity are predominantly observed within the first few months following moderate to severe TBI (e.g., Bonnelle et al., 2012; Caeyenberghs et al., 2012; Castellanos et al., 2011; Hillary et al., 2011; Nakamura, Hillary, & Biswal, 2009). This pattern of hyperconnectivity has been hypothesized to reflect a compensatory mechanism to increase neural resource utilization and re‐establish network‐level communication following severe disruption (i.e., so‐called “Hyperconnectivity Hypothesis” [Hillary & Grafman, 2017; Hillary et al., 2015]) and may underlie more complex pathophysiological and clinical features associated with TBI compared with subconcussive RHIs.

Reduced connectivity strength between the PCC and short‐ and long‐range afferents of the DMN may derive from reduced metabolism (Nakashima et al., 2007), reduced cerebral blood perfusion (Kim et al., 2010), or diffuse axonal injury (Bonnelle et al., 2011; Kinnunen et al., 2011) of the PCC. Recent studies have also demonstrated an increase in neurofilament light (NF‐L) following subconcussive head impacts (Rubin et al., 2019; Shahim, Zetterberg, Tegner, & Blennow, 2017), which has been interpreted to be a reflective marker of axonal white matter injury (Shahim et al., 2016; Zetterberg, Smith, & Blennow, 2013). Previously, our group has demonstrated abnormalities in DTI‐derived white matter metrics following subconcussive RHI exposure in youth and high‐school football players (Bahrami et al., 2016; Davenport et al., 2014; Davenport et al., 2016). Given the strong relationship between structural and functional connectivity indices (Damoiseaux & Greicius, 2009; Greicius, Supekar, Menon, & Dougherty, 2009; Straathof, Sinke, Dijkhuizen, & Otte, 2019), it is possible that diffuse injury to white matter microstructure following RHI exposure leads to transient hypoconnectivity of DMN architecture.

The underlying changes in DMN FC following subconcussive RHI exposure in collision‐sport athletes with respect to a control group has not been well characterized. A study in collegiate female rugby players demonstrated a DMN hyper‐connectivity pattern with the PCC at both the start and 2–3  months following the season compared with non‐collision sport controls (Manning et al., 2020). The respective hypo‐ versus hyper‐connectivity pattern between our work and their study could be explained by several factors, including age, gender, and inter‐sport differences in head impact dynamics. One study using a whole‐brain correlation approach in high‐school football players reported both an increase and decrease in the number of significant positive connections with the PCC/precuneus at several time points during the season compared with a baseline control value, including an increase in postseason connections (Abbas, Shenk, Poole, Breedlove, et al., 2015). While this study provides a perspective on the total number of regional correlations between the PCC and anatomical parcellations within and beyond the DMN, our work characterized group differences with respect to the relative strength of functional connections within a specific set of network hubs that consistently show a positive correlation with the PCC under task‐negative conditions (Fox et al., 2005; Greicius et al., 2003).

While it may be unlikely that a single season of youth football RHI exposure leads to adverse long‐term clinical outcomes observed for former career football players (McKee et al., 2009; Mez et al., 2017; Roberts et al., 2019; Stamm et al., 2015), it is also important to recognize the potential implications. Koerte et al. (2017) demonstrated that soccer players exposed to RHIs from ball heading did not improve in executive control performance during the season akin to their non‐contact sport control counterparts. This was posited to reflect suppressed developmental benefit in athletes exposed to RHIs. This finding is consistent with another study that demonstrated impaired oculomotor executive control function following mTBI in a college‐aged cohort (Webb, Humphreys, & Heath, 2018). Functional abnormalities within the PCC and DMN are characteristic of a broad spectrum of neurological and psychiatric conditions (Buckner et al., 2008; Leech & Sharp, 2014; Zhang & Raichle, 2010). Abnormal DMN function has been shown to be related to cognitive impairment following neurotrauma. For example, Mayer et al. (2011) demonstrated that DMN FC predicted cognitive complaints during the sub‐acute phase following a concussion. Moreover, attentional and information processing task performance has been shown to correlate with DMN FC following TBI (Bonnelle et al., 2011; Sharp et al., 2011). It remains unclear, however, whether subconcussive head impacts, independent of concussive head injury, represent a catalyst for long‐term clinical impairment. Future, large‐scale, multimodal, and longitudinal studies will be critical to determining the neuronal mechanisms underlying abnormal FC changes in youth tackle football players, as well as the potential long‐term neurological outcomes.

### 4.2 Longitudinal DMN connectivity changes in football players

A subsample of football players with off‐season follow‐up data (Cohort 2) provided the opportunity to examine whether in‐season delta FC, representing a period of RHI exposure, differed from that of off‐season delta FC, representing a 7‐month withdrawal from football‐related activity. Regions identified as displaying significant group‐level in‐season delta FC effects in the ROI analysis were decomposed longitudinally in Cohort 2 football participants (i.e., in‐season vs. off‐season). The results showed that in‐season delta FC yielded a negative mean across all ROIs in football players, whereas an opposite compensatory effect was observed for off‐season delta FC (Figure 5).

The negative directional change between preseason and postseason FC of the DMN is in agreement with prior work examining changes in DMN FC following short‐ and long‐term exposure to subconcussive RHIs. Johnson et al. (2014) reported a decrease in short‐range connectivity between the PCC and retrosplenial cortex following a single game session in collegiate rugby players. Similarly, football players here demonstrated a negative in‐season delta FC mean for the PCC/precuneus ROI, which also encompasses the portions of the retrosplenial cortex. Slobounov et al. (2017) reported a shift from a pattern of preseason positive correlation to postseason anti‐correlation between the isthmus of the cingulate cortex and a voxel cluster comprising the fusiform/middle occipital gyri in collegiate football players. In a pair of studies in high‐school football players, Abbas and colleagues reported a reduced number of regional correlations between the central PCC/precuneus node of the DMN and whole‐brain anatomical parcellations at postseason compared with preseason (Abbas, Shenk, Poole, Breedlove, et al., 2015; Abbas, Shenk, Poole, Robinson, et al., 2015).

The positive change in off‐season delta FC is in slight contrast to the work by Abbas, Shenk, Poole, Robinson, et al. (2015), which reported a reduced number of whole‐brain connections with the PCC at initial and 6‐month postseason time points relative to preseason. This was interpreted to reflect a long‐term repair process underlying mechanical stress of cumulative RHI exposure. This is consistent with prior work showing persistent long‐term reduced DMN FC in collegiate football players (Zhu et al., 2015) and adults (Mayer et al., 2011) following a concussion. Our findings do not support a long‐term outcome, as regions displaying a negative in‐season change in FC encountered a positive rebound effect during the 7‐month off‐season interval. This discrepancy could be attributed to methodological differences. Abbas, Shenk, Poole, Robinson, et al. (2015) examined the total number of regional connections between the PCC and whole‐brain anatomical parcellations, whereas our work examined the relative FC strength of within‐network DMN architecture. An alternative view is that additional accrual of RHIs with increasing levels of play (Broglio et al., 2009; Broglio et al., 2011; Broglio, Surma, & Ashton‐Miller, 2012; Daniel et al., 2012; Kelley et al., 2017; Urban et al., 2013; Urban et al., 2019) leads to more consequential long‐term recovery outcomes. It is possible that reduced accumulation of head impacts over a shorter participation duration in youth players better supports off‐season compensation of dysfunctional connectivity patterns. In support of this interpretation, a subsequent study in high‐school players by Abbas, Shenk, Poole, Robinson, et al. (2015) reported a deviation in DMN connections from control values even at preseason. In contrast, football and control participants in the present study yielded reasonably consistent preseason FC values, demonstrating no significant group effects in the voxel‐wise analysis and only in one of 12 regions in the ROI analysis.

It is important to note that despite the directional change between in‐ and off‐season delta FC values, no statistically significant main effects of time were observed, indicating that the negative in‐season delta FC did not reliably differ from the positive off‐season delta FC across ROIs. This may be because the sample size for Cohort 2 was small compared with the original Cohort 1, leading to high intersubject variability in mean in‐ and off‐season delta Z‐score values across ROIs. Furthermore, while it is reasonable to assume that levels of RHI exposure declined following off‐season withdrawal from play, football players were not adequately monitored for head impact exposure outside of football during off‐season months. Eight of the 17 football players in Cohort 2 reported participation in off‐season sports, including basketball and soccer. It is possible that continued participation in activities with risk for head impacts during off‐season months in some players led to an attenuation of apparent off‐season positive delta FC values. Future work including larger sample size and improved control of off‐season participation variables (i.e., complete refrain from sport) will better determine the longitudinal outcomes of RHI exposure in youth players.

### 4.3 Effects of head impact frequency on functional connectivity outcomes

A final key issue to address is whether the number of subconcussive head impacts in youth football players differentially influenced DMN FC outcomes. Subconcussive head impacts have been historically overlooked because they do not evoke clinically recognizable symptoms. In recent years, however, it has become increasingly clear that subconcussive RHIs may have short‐ and long‐term neurological consequences. Abnormal diffusion tensor imaging (DTI) indices of white matter microstructure have been shown following single‐ and multi‐season exposure to subconcussive head impacts (Koerte et al., 2012; Koerte, Ertl‐Wagner, Reiser, Zafonte, & Shenton, 2012; Manning et al., 2020; Mayinger et al., 2018). Diffusion MRI studies incorporating the HIT system have pointed to an association between the number and severity of subconcussive head impact exposure and abnormal brain microstructure in youth, high‐school, and collegiate football players (Bahrami et al., 2016; Bazarian et al., 2014; Chun et al., 2015; Davenport et al., 2016; McAllister et al., 2014). Aside from acute changes, age at first exposure (≤ 12 years) to RHIs in professional football players may be associated with long‐term damage of commissural white matter and more severe neurobehavioral impairment (Alosco et al., 2018; Stamm, Bourlas, et al., 2015; Stamm, Koerte, et al., 2015). Additionally, widespread deposition of hyperphosphorylated tau as neurofibrillary tangles, the pathological hallmark of CTE, is associated with prolonged exposure to head impacts, irrespective of clinical concussion (McKee et al., 2013; Stein, Alvarez, & McKee, 2015; Tagge et al., 2018). Taken together, this evidence underscores the critical importance of more focused advanced imaging studies characterizing the effects of subconcussive RHIs on underlying brain pathophysiology, especially in vulnerable youth collision‐sport athletes. A deeper understanding of the relation between head impact exposure and FC outcomes may contribute to refined sport safety policies and targeted intervention techniques to counter possible neurological detriments.

As shown in Table 1, current youth football players experienced a median of 257 head impacts (median bootstrapped 95% confidence: 161–383) over the course of the season, with median linear and rotational peak resultant acceleration values of 18.3 g and 925 rad/s^2^, respectively. Importantly, football players yielded a low median RWE_CP_, indicating that FC outcomes reflect the consequence of head impacts on a subconcussive scale with low concussion risk. We were interested in determining whether a high and a low number of head impacts differentially influenced the change between preseason and postseason FC in Cohort 1 and between in‐season and off‐season delta FC in Cohort 2. In Cohort 1, low impact players revealed no differences between preseason and postseason across all ROIs, whereas high impact players revealed reduced postseason FC compared with preseason in the r‐ and l‐LTC (Figure 6a). Several other regions in the high impact group, but not the low impact group, also showed a trend towards significantly reduced postseason FC. Similarly, low impact players in Cohort 2 revealed no time effects between in‐season and off‐season delta FC across ROIs, whereas the positive off‐season delta FC was significantly increased compared with the negative in‐season delta FC mean of the PFC/l‐DFC, r‐DFC, r‐TPC, r‐ and l‐LTC, and the global DMN in high impact players (Figure 6b).

The results from this analysis provide initial evidence that the number of experienced head impacts at the youth football level may be a key contributing factor to abnormal DMN FC outcomes. It is possible, however, that other factors contributed to the FC disparity between low and high impact groups. As shown in Table 1, high impact players in Cohort 1 were also characterized by a significant increase in median peak resultant linear acceleration and RWE_CP_. A key aim for future work should include combined consideration for both the number and severity of head impacts on FC outcomes. Positional play is also associated with differences in head impact exposure, with fundamental tackle positions (i.e., linemen, linebackers) engaging in more frequent collisions compared with skilled ball‐handling positions (Broglio et al., 2009; Crisco et al., 2010; Crisco et al., 2011). Youth football players in the current study were characterized by variable offensive and defensive positional play and subject to recurrent positional substitutions throughout the course of practice and game sessions. Thus, the impact of positional play on FC outcomes could not be adequately addressed in the present youth players. It is also important to consider the effect of covariates such as BMI on FC outcomes. Despite variable positional play, larger players may be more favorably positioned in fundamental tackle positions and engage in more frequent collisions. There is also evidence suggesting a relationship between BMI and DMN FC (Kullmann et al., 2012). Players in the high impact group for Cohort 1 (BMI = 21.3 [*SD* = 2.3]) showed a trend towards increased BMI compared with the low‐impact group (BMI = 19.7 [*SD* = 2.5]) (two‐sample *t* test, *p* = .054). Thus, BMI may have had an effect on the relationship between head impact frequency and the changes in DMN FC between preseason and postseason. In Cohort 2, BMI did not reliably differ between players in the high (BMI = 21.3 [*SD* = 2.3]) and low impact (BMI = 20.5 [*SD* = 4.4]) groups (two‐sample *t* test, *p* = .65), suggesting that BMI did not have an effect on the relationship between head impacts and the change between in‐season and off‐season FC delta.

The results from this analysis emphasize the importance of limiting head impacts in youth football and support implementation of targeted rule interventions and policy changes to reduce the number of sustained head impacts in players (Kerr et al., 2015; Ocwieja et al., 2012). These results may also serve to inform parental decision‐making regarding youth football participation. Many parents are in support of age‐related restrictions on tackling (Chrisman et al., 2019) and have concerns about later‐life neurological detriments stemming from their children sustaining football‐related head injuries (Kroshus, Bowen, Opel, Chrisman, & Rivara, 2020). Certainly, this perspective is understandable given widespread media attention surrounding studies with overwhelming evidence of CTE in former professional football players (Mez et al., 2017). It is important to emphasize, however, that FC changes and associated short‐term and long‐term outcomes related to subconcussive RHIs, especially in youth collision sport athletes, are not yet fully understood. Future longitudinal studies will play an important role in shaping our perspectives regarding the social benefits and safety risks associated with youth football participation.

### 4.4 Limitations

There are several important limitations of the current study. First, the current seed‐based methodology does not account for possible widespread network‐level responses to RHIs across other intrinsic RSNs previously shown to be affected by sport‐related neurotrauma (Bharath et al., 2015; Mayer, Bellgowan, & Hanlon, 2015). Future studies implementing a more comprehensive exploratory approach (e.g., independent component analysis [e.g., Calhoun & Adalı, 2012]) are important to better characterize intrinsic functional architecture changes underlying RHI exposure in youth sport cohorts. Second, while the initial and follow‐up scans for players coincided with the preseason and postseason for football players, the initial and follow‐up scans did not always coincide with the preseason and postseason of the respective sport for controls. Although the average inter‐scan MRI acquisition interval was consistent for both football and control groups (i.e., ~ 4 months), the DMN connectivity profile of control participants could have been influenced by the absence of sport‐related involvement between initial and follow‐up scans. Third, the HIT System used for acquisition of head impact kinematic data is associated with some individual impact detection and acceleration measurement error. However, this device limitation has been shown to be comparable with other head impact devices and well within an acceptable range of error (Beckwith et al., 2012). A fourth limitation is the arbitrary grouping of high‐ and low‐impact groups while examining the effects of head impact frequency on DMN FC outcomes. For Cohort 1, the upper and lower 30th percentile of the head impact frequency distribution was used to separate players, while players falling between those cutoff points were removed. This procedure was used to provide adequate separation of players corresponding to the head impact frequency distribution, as well as an adequate sample size within each impact partition. For Cohort 2, the 50th percentile was used in order to limit the exclusion of players from an already small pool of participants. An important step for future work may include a sensitivity analysis to determine the number of head impacts required to induce network‐level changes in FC. Lastly, it is important to emphasize the exploratory nature of the analyses for Cohort 2 due to its relatively small sample size.

## 5 CONCLUSION

This study represents initial evidence in a high‐risk youth collision‐sport cohort that a single season of subconcussive head impact exposure in the absence of concussion causes reduced network‐level FC of widespread DMN regions compared with non‐collision sport controls. In the longitudinal analysis, in‐season delta FC was characterized by a negative directional change between preseason and postseason, whereas an opposite, compensatory effect was observed for off‐season delta FC between postseason and follow‐up. Lastly, the number of experienced head impacts in youth football players proved to be a key contributing factor to FC alterations. These findings extend evidence from other neuroimaging modalities and advance our understanding of the underlying pathophysiology characterizing subconcussive head impact exposure in youth football players.

## CONFLICT OF INTERESTS

The authors declare no competing financial or non‐financial interests.

## Supporting information


**Appendix S1:** Supporting information

## Data Availability

De‐identified data are available upon reasonable request from the corresponding author.
