# Supplementary material for: Mapping default mode connectivity alterations following a single season of subconcussive impact exposure in youth football
Source: Hum Brain Mapp. 2021 Mar 18;42(8):2529–45. doi: 10.1002/hbm.25384 (PMC8090779; doi:10.1002/hbm.25384)
Supplement: Supplementary file 1 — Appendix S1: Supporting information [file HBM-42-2529-s001.docx]

| **Table 1a. Football Participants** | | | |  |  |  |  |  |
| --- | --- | --- | --- | --- | --- | --- | --- | --- |
| **Cohort** | **Age** | **Height (In)** | **Weight (lbs)** | **BMI** | **Race** | **Contact Sport Years** | **Offseason Sport(s)/ Previous Sport(s)** | **Previous Sport(s)** |
| 1 | 12 | 60.00 | 92.8 | 18.12 | African American | 8 | Basketball | Basketball |
| 1 | 11 | 58.75 | 113.6 | 23.14 | African American | 5 |  |  |
| 1 | 12 | 60.50 | 129.8 | 24.93 | African American | 6 |  | Lacrosse |
| 1 | 13 | 67.25 | 129.2 | 20.08 | African American | 5 | Basketball | Basketball |
| 1 | 11 | 56.50 | 91.6 | 20.17 | African American | 3 |  |  |
| 1 | 12 | 60.00 | 87.8 | 17.15 | African American | 0 |  |  |
| 1 | 12 | 59.62 | 133.2 | 26.34 | African American | 5 | Basketball, Track | Basketball, Track |
| 1 | 12 | 62.25 | 102.8 | 18.65 | Caucasian | 5 | Lacrosse | Lacrosse |
| 1 | 13 | 67.00 | 116.2 | 18.20 | Declined | 7 | Basketball, Soccer | Basketball, Soccer |
| 1 | 11 | 61.25 | 112.8 | 21.14 | African American | 5 | Basketball, Soccer | Basketball, Soccer |
| 1 | 11 | 62.25 | 102.4 | 18.58 | African American | 0 |  |  |
| 1 | 12 | 60.00 | 111.2 | 21.71 | African American | 6 | Basketball | Basketball |
| 1 | 12 | 64.00 | 163.4 | 28.04 | African American | 4 |  | Basketball |
| 1 | 12 | 58.50 | 87.4 | 17.95 | Caucasian | 3 | Lacrosse | Lacrosse |
| 1 | 14 | 64.75 | 110.2 | 18.48 | African American | 9 | Baseball | Baseball |
| 1 | 12 | 56.25 | 95.8 | 21.29 | African American | 6 |  |  |
| 1 | 12 | 57.63 | 90.4 | 19.13 | African American | 5 |  |  |
| 1 | 11 | 55.38 | 72.8 | 16.69 | African American | 1 |  |  |
| 1 | 12 | 69.00 | 160.2 | 23.65 | African American | 6 | Basketball, Track | Basketball, Track |
| 1 | 10 | 57.00 | 98.6 | 21.33 | African American | 5 | Basketball, Lacrosse | Basketball, Lacrosse |
| 1 | 12 | 61.00 | 102.2 | 19.31 | African American | 5 |  |  |
| 1 | 13 | 60.50 | 128.4 | 24.66 | African American | 3 |  |  |
| 1 | 13 | 64.00 | 123.0 | 21.11 | African American | 5 |  |  |
| 1 | 13 | 63.50 | 131.6 | 22.94 | Caucasian | 1 | Taekwondo | Taekwondo |
| 1 | 14 | 66.75 | 140.4 | 22.15 | African American | 5 |  |  |
| 1 | 11 | 53.75 | 62.0 | 15.09 | African American | 6 | Basketball | Basketball |
| 1 | 10 | 58.00 | 105.6 | 22.07 | African American | 5 |  |  |
| 1 | 11 | 60.25 | 104.0 | 20.14 | African American | 1 |  |  |
| 1 | 10 | 60.25 | 110.6 | 21.42 | African American | 5 |  |  |
| 1 | 10 | 54.75 | 86.2 | 20.22 | African American | 2 |  |  |
| 1 | 10 | 59.75 | 114.0 | 22.45 | Caucasian | 3 |  |  |
| 1 | 10 | 60.50 | 85.6 | 16.44 | African American | 5 | Basketball, Spring Tackle Football | Basketball |
| 1 | 11 | 58.75 | 82.0 | 16.70 | African American | 5 | Track | Track |
| 1,2 | 11 | 63.00 | 100.4 | 17.78 | African American | 6 |  | Basketball, Flag Football |
| 1,2 | 9 | 59.25 | 106.2 | 21.27 | African American | 5 |  |  |
| 1,2 | 12 | 57.75 | 88.4 | 18.63 | African American | 5 |  |  |
| 1,2 | 10 | 57.25 | 88.2 | 18.92 | African American | 3 | Basketball, Track | Basketball, Track |
| 1,2 | 11 | 59.25 | 109.6 | 21.95 | African American | 4 | Basketball | Basketball |
| 1,2 | 11 | 58.25 | 88.6 | 18.36 | Declined | 3 |  |  |
| 1,2 | 12 | 61.00 | 123.4 | 23.31 | African American | 6 |  |  |
| 1,2 | 12 | 64.12 | 149.0 | 25.48 | African American | 5 |  |  |
| 1,2 | 13 | 60.38 | 103.2 | 19.90 | African American | 6 |  |  |
| 1,2 | 13 | 66.00 | 130.4 | 21.04 | African American | 5 | Baseball, Basketball, Golf | Baseball, Basketball, Golf |
| 1,2 | 10 | 56.00 | 74.0 | 16.59 | African American | 5 | Basketball | Basketball |
| 1,2 | 12 | 60.25 | 144.8 | 28.04 | Caucasian | 1 |  |  |
| 1,2 | 10 | 56.25 | 104.8 | 23.28 | African American | 2 | Basketball | Basketball |
| 1,2 | 12 | 62.50 | 138.8 | 24.98 | African American | 1 |  |  |
| 1,2 | 10 | 57.75 | 93.6 | 19.73 | African American | 4 | Basketball | Basketball |
| 1,2 | 12 | 60.89 | 83.6 | 15.85 | African American | 5 | Basketball, Soccer, Taekwondo, Track | Basketball, Soccer, Taekwondo, Track |
| 1,2 | 11 | 57.25 | 103.0 | 22.09 | Declined | 3 | Basketball, Soccer | Basketball, Soccer |

| **Table 1b. Control Participants** | | |  |  |  |
| --- | --- | --- | --- | --- | --- |
| **Age** | **Height (In)** | **Weight (lbs)** | **BMI** | **Race** | **Sport** |
| 14 | 60.50 | 90.6 | 17.40 | Caucasian | Baseball |
| 13 | 64.25 | 117.6 | 20.03 | Caucasian | Baseball |
| 12 | 60.00 | 118.4 | 23.12 | Caucasian | Baseball |
| 11 | 56.25 | 75.2 | 16.71 | Caucasian | Baseball |
| 12 | 57.25 | 84.2 | 18.06 | Caucasian | Baseball |
| 8 | 51.50 | 76.0 | 20.14 | Caucasian | Baseball |
| 12 | 54.75 | 80.6 | 18.90 | Caucasian | Baseball |
| 10 | 55.00 | 73.2 | 17.01 | African American | Basketball |
| 13 | 66.00 | 109.4 | 17.66 | Caucasian | Basketball |
| 10 | 52.50 | 62.6 | 15.97 | Caucasian | Basketball |
| 12 | 58.50 | 77.2 | 15.86 | African American/Caucasian | Basketball |
| 10 | 53.00 | 68.4 | 17.12 | African American | Basketball |
| 12 | 62.00 | 107.8 | 19.71 | Caucasian | Basketball |
| 11 | 60.00 | 80.2 | 15.66 | African American | Basketball |
| 8 | 52.75 | 56.8 | 14.35 | Caucasian | Soccer |
| 11 | 57.50 | 67.8 | 14.42 | Caucasian | Soccer |
| 13 | 61.50 | 99.2 | 18.44 | Caucasian | Tennis |
| 12 | 64.25 | 109.6 | 18.66 | Caucasian | Tennis |
| 11 | 59.50 | 90.6 | 17.99 | Caucasian | Karate |
| 12 | 56.75 | 65.6 | 14.32 | Caucasian | Swimming |
